# The spectrosome of occupational health problems

**DOI:** 10.1371/journal.pone.0190196

**Published:** 2018-01-05

**Authors:** Delphine Bosson-Rieutort, Régis de Gaudemaris, Dominique J. Bicout

**Affiliations:** 1 Grenoble Alpes University / CNRS / TIMC-IMAG UMR 5525 (EPSP team - Environment and Health Prediction of Populations), Grenoble, France; 2 Occupational and Environmental Diseases Centre, Grenoble Teaching Hospital (CHU Grenoble), Grenoble, France; 3 Biomathematics and Epidemiology EPSP-TIMC, VetAgro Sup, Veterinary Campus of Lyon, Marcy l’Etoile, France; 4 Laue - Langevin Institute, Theory Group, France; Stony Brook University, Graduate Program in Public Health, UNITED STATES

## Abstract

Given the increased prevalence of cancer, respiratory diseases, and reproductive disorders, for which multifactorial origins are strongly suspected, the impact of the environment on the population represents a substantial public health challenge. Surveillance systems have become an essential public health decision-making tool. Networks have been constructed to facilitate the development of analyses of the multifactorial aspects of the relationships between occupational contexts and health. The aim of this study is to develop and present an approach for the optimal exploitation of observational databases to describe and improve the understanding of the (occupational) environment–health relationships, taking into account key multifactorial aspects. We have developed a spectral analysis (SA) approach that takes into account both the multi-exposure and dynamic natures of occupational health problems (OHPs) and related associations. The main results of this paper are to present the construction method of the “spectrum” and “spectrosome” of OHPs (range and structured list of occupational exposures) and describe the information contained therein with an illustrative example. The approach is illustrated using the case of non-Hodgkin lymphoma (NHL) from the French National Occupational Diseases Surveillance and Prevention Network database as a working example of an occupational disease. We found that the NHL spectrum includes 40 sets of occupational exposures characterized by important multi-exposures, especially solvent combinations or pesticide combinations, but also specific exposures such as polycyclic aromatic hydrocarbons, formaldehyde and ionizing radiation. These findings may be useful for surveillance and the assessment of occupational exposure related to health risks.

## Introduction

The impacts of the environment on the population are obvious and represent a substantial public health challenge. Since the 2000s, there has been an increase in the prevalence of cancer, respiratory diseases, and reproductive disorders, for which multifactorial origins are strongly suspected. Likewise, occupational diseases and potentially associated exposures represent a major public health issue for identifying and preventing new threats to workers’ health. In the normal course of their activities in various occupational contexts, many workers are subjected to physical, chemical or biological exposures that may have an impact on the development of pathologies; furthermore, working conditions and occupational exposures are rapidly changing. In France, for example, approximately 50 000 new occupational diseases are recognized by the Worker’s Compensation Act, mostly in the industrial sector [1], and 4 to 8.5% of cancer could be attributable to occupational exposures [2]. However, depending on the type of cancer, this percentage could be much higher. For example, 10 to 15% of the lung cancer cases among men in the USA could be attributable to the occupational environment [3]. Between 5 and 25% [4] of bladder cancer cases may be attributed to occupational exposures, depending on the country, with an estimate of 10% in the USA [5] and 8% in Europe [6]. The portion of leukemia cases attributable to occupational exposures is estimated to be 10% in the USA among men [3], 5% in Europe [7] and 18.5% in Finland [8].

In these circumstances, surveillance systems are an essential public health decision-making tool. Surveillance systems need to be developed based on analyses of the multifactorial aspects of the relationships between occupational contexts and health. The object of interest in such surveillance is the occupational health problem (OHP) that can be regarded as a diagnosed disease associated with occupational exposures within occupational contexts for which one or several exposures are potentially causative. From this perspective, networks (and associated databases) have been constructed to allow developing and monitoring, such as The Health and Occupation Research Network (THOR) in the UK [9], the French National Occupational Diseases Surveillance and Prevention Network (RNV3P) in France [10], the “Malattie Professionali” (occupational disease) surveillance system (MALPROF) in Italy [11] and IDEWE in Belgium.

Within the framework described above, our aim was to develop an approach allowing an optimal exploitation of databases for analyzing, characterizing, describing and, improving our understanding of (occupational) environment–health relationships, accounting for multifactorial aspects. We have developed a spectral analysis (SA) approach that takes into account the multi-exposure and dynamic natures of OHPs and related associations. Our approach was inspired by the exposome approach [12,13] and consists of constructing and describing the time-varying occupational exposure spectra of OHPs leading to spectrosomes. An OHP spectrosome, representing the signature of the association between a disease and a set of occupational exposures, consists of *i*) a spectrum, range or structured list of occupational exposures, and *ii*) a dynamic relational network between spectrum elements. This paper presents a method for constructing the spectrosomes of OHPs and describes the information contained in a spectrosome. For the sake of description and illustration of the approach, we used the data from the RNV3P database [10] and used as a working example of occupational disease the case of non-Hodgkin lymphoma (NHL), a cancer with an incidence that has been increasing since the 1970s and for which risk factors are not yet well-known [14–17]. We found that the NHL spectrum includes 40 sets of occupational exposures, characterized by important multi-exposures, especially solvent combinations or pesticide combinations, but also specific exposures such as polycyclic aromatic hydrocarbons, formaldehyde and ionizing radiation. These findings may be useful for surveillance and the assessment of occupational exposure related to health risks.

## Methods: The spectral analysis approach

The main objective of the SA approach is to enable an optimal use of large-scale observational databases, taking into account time and the multiplicity of causes leading to the appearance of an event of interest. Based on the exposome approach [13], the main steps of this analysis are *i)* to identify, from a sample of data, all potential events of interest (modalities) related to a target variable, *ii)* to determine the potential associations between these events as “motifs” of interest and *iii)* to apply the different indicators of SA to characterize the set of motifs in terms of importance, specificity and dynamic status.

**Fig 1:**
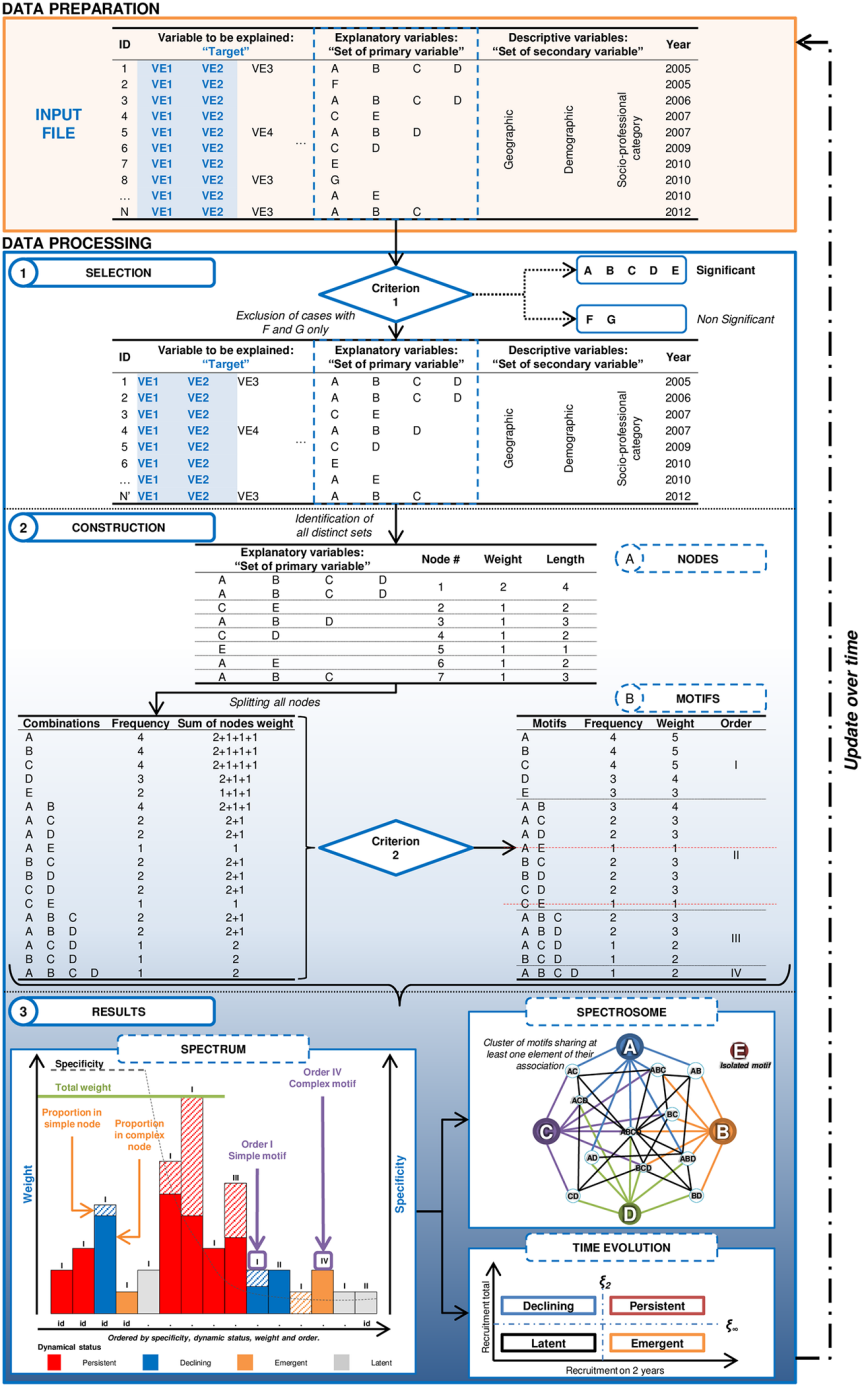
Description of the spectral analysis approach: Data preparation and data processing using this approach. The first group corresponds to data preparation with the schematic structure of an observational database used as the input file. The second group corresponds to data processing with 1/ selection of significant observations based on significant criteria applied to modalities; 2/ construction of nodes and motifs of the exposome and spectrosome; and 3/ the main results of the spectral analysis represented by the spectrum and the spectrosome over time.

Fig 1 describes all of the steps in implementing the SA from a more general perspective.

### Data preparation

Observational databases are organized in a table that consists of observations (cases) in rows and variables in columns. A minimal sample of input data contains (Fig 1, “input file”) the variable of interest (the target) plus one or more explanatory variable(s) related to the target, which will be considered as a set of primary variables; and a time variable allowing for the dynamic part of the analysis (Fig 1, “data preparation”). Descriptive variables such as geographic or demographic variables could be considered optional. We will refer to distinct values of a variable as “modalities”. For example, in Fig 1 the target variable has 4 distinct modalities: “VE1”, “VE2”, “VE3” and “VE4” and the explanatory variables have 7 distinct modalities: “A”, “B”, “C” …, “G”. The variables should be standardized using a thesaurus or nomenclature allowing the use of codes and avoiding syntax errors.

### SA process

#### Selection

To select observations to be considered for further analyses, the target should be specified on the basis of a set of modalities, such as “VE1” and “VE2” in Fig 1. Next, all modalities of the explanatory variables are screened and those that are significantly relevant and potentially related to the target are listed (criterion 1). Selection criteria can be based on the frequency of the modality or on a complementary variable (such as “imputability”) indicating the strength of the association between the event and each modality recorded in explanatory variables. Only observations with at least one significant modality are retained for the formation of the exposome nodes, as introduced and discussed by Faisandier et al. [13].

**Selection criterion based on frequency:** The frequency-based significance for a modality *i* uses the relative risk ${RR}_{i}=n_{i,\Delta t}/\frac{1}{H}\sum_{j=1}^{H}n_{j,\Delta t}$, where *H* is the total number of distinct modalities and *n*_*i*,Δ*t*_ is the number of observations with the modality *i* recruited (recorded) within the time period Δ*T*. This number can be obtained as $n_{i,\Delta t}=\frac{n_{i}}{T}\Delta t$, where *n*_*i*_ is the total recruitment number over the entire period *T* of the study (or reference period). Based on the significant score at 95% for a normally distributed reduced (mean/standard deviation) variable, a modality *i* is considered significant when *RR*_*i*_ > 1.96.

**Selection criterion based on imputability:** When the imputability is available, the number *n*_*i*_ of observations with the modality *i* is associated with a distribution of imputability. In this case, the p-value *p*_*i*_ for each modality *i* can be used as a significant criterion. Let *Q*_*k*_ denote the probability of obtaining *k* recruitments of a modality within the sample as $\sum_{k=0}^{\infty }Q_{k}=1$. The probability *p*_*i*_ of obtaining more than *n*_*i*,Δ*t*_ recruitment of the modality *i* within the time period Δ*T* is given by: $p_{i}=P(\geq n_{i,\Delta t})=\sum_{k=n_{i,\Delta t}}^{\infty }Q_{k}=1-\sum_{k=0}^{n_{i,\Delta t}-1}Q_{k}$. And, the recruitment is considered as not random and the modality *i* is then significant when *p*_*i*_ ≤ *α* (α is currently set to 5%).

For the RNV3P database, *Q*_*k*_ is assumed to follows a Poisson distribution of parameter *λ*, empirically related to the imputability *I* by: $\lambda =1.37-1.32\sqrt{I}$, with 0 ≤ *I* ≤ 3.

#### Construction of significant motifs

Data that met criterion 1 were restructured as a network “exposome” as described in Faisandier *et al*. [13]. A node *ν* of the exposome network corresponds to a set of observations sharing exactly the same set of modalities. The number of identical set corresponds to the node’s weight *a*_*v*_ and the number of modality in the set corresponds to the node’s length. For example, in Fig 1 (step 2 “construction”), node #1 “A/B/C/D” has 2 copies in the database and a length equal to 4.

Restructuring the dataset in a structural network enables the consideration of the multiplicity of causes (modalities) and highlighting potential links between them. Depending on its length (number of modalities in the set) each node *ν* is split to generate all potential combinations of modalities as Cν=∑i=1d(di) as illustrated below in Fig 1 and Supplemental S1 Fig. Significant combinations are considered as the “significant motifs” related to the target variable if $q_{i}=\sum_{\nu =1}^{V}{\left(a_{c,\nu }\right)}^{2}>3$ (criterion 2).

#### Spectral analysis

Significant motifs are characterized using various indicators which will be used in spectrum and spectrosome, such as weight, order, specificity and dynamic status (Fig 1, step 3 “results”):

- **Weight:** For a motif *m*, the weight *w* corresponds to the total number of recruitment(s) on the selected sample, represented by the total number of cases or the sum of the weight of the nodes.
- **Order:** Number of modalities recorded in the same motif: 1 modality as Order I (“A”), 2 modalities as Order II (“A/B”), 3 modalities as Order III (“A/B/C”), etc. Motifs with Order > 1 contain multi-information related to the target variable.
- **Specificity:** Ranging between 0 and 1, specificity allows for the characterization of the composition of each motif. A specificity S = 1 implies that the motif is specific to a particular node, essentially recorded in the same motif; S = 0 corresponds to a ubiquitous motif found in several distinct nodes or combinations (S2 Fig). Inspired by the Shannon information index [18], specificity is defined as:
  $$S_{m}=\frac{H_{max}-H_{m}}{H_{max}-H_{min}}$$
  where $H_{m}=-\sum_{\nu =1}^{u_{m}}\left(\frac{a_{m,v}}{w_{m}}\times \text{log}\;\frac{a_{m,v}}{w_{m}}\right)$ is the Shannon entropy calculated on the number of nodes containing the motif, with its minimum and maximum *H*_*m*,*max*_ = log(*u*_*m*_) and $H_{m,min}=\text{log}\;\left(\frac{w_{m}}{w_{m}-u_{m}+1})+(\frac{u_{m}-1}{w_{m}}\times \text{log}\;\left(w_{m}-u_{m}+1\right)\right)$, respectively.
  As a reminder, *w* corresponds to the motif’s weight, *a* to the node’s weight and *u* to the number of nodes containing the motif.
- **Dynamic status** (Fig 1, in step 3 “time evolution”): Based on the Reporting Index (RI) representing the proportion of the motif relative to the other) and using a threshold *ξ*_*R*_, four dynamic profiles are defined: persistent, declining, emergent, and latent. The RI in the time frame from t_0_ to t is calculated as follows:
  RIm(t|t0)=∑=t0twm(i)∑n=1M(∑i=t0twn(i))
  Defining the threshold as ξR=1∑m=1M[RIm(t|t0)]2, which corresponds to the minimal number of motifs for explaining the essential recruitment in the given period, two limits are used to define the status: ${RI}_{\infty ,m}={\text{lim}}_{t_{0}\to -\infty }\;RI(t|t_{0})$ and *RI*_2,*m*_ = *RI*(*t* | *t* − 1). Therefore, a motif is persistent when it is substantially recorded during both the entire period and the 2-year interval (red on Fig 1), a declining motif is substantially recorded during the entire period but much less during the 2-year interval (blue on Fig 1), an emergent motif is weakly reported during the entire period but much more during the last two years (orange in Fig 1), and a latent motif is weakly recorded during both the entire period and the 2-year interval (gray in Fig 1).

The two main output figures of the spectral analysis are the spectrum, which is the signature of the analysis, summarizing all indicators of SA, and the “spectrosome”, which we define as the unpacked spectrum, illustrating the complexity of the structure and relationship between highlighted motifs. The spectrosome is decomposed into isolated motifs and imbricated clusters, where a cluster is a fully connected network formed at most of a single motif of order I connected with motifs of higher orders.

## Illustrative results

To specify and illustrate the SA approach outlined above, we consider the case of non-Hodgkin lymphoma (NHL), a cancer with an incidence that has been increasing since the 1970s and with risk factors that are not yet well-known [14–17]. Within the framework of this study as explained in the Methods section and in Fig 1, we used as the input file the database from the French National Occupational Diseases Surveillance and Prevention Network (RNV3P) within which the target and set of explanatory variables are “work related NHL cases” and “occupational exposures”, respectively.

Briefly, the RNV3P network was created in 2001 and records in a systematic and standardized way all patients’ cases diagnosed with diseases potentially related to occupational exposures. During a consultation or occupation interview, the network’s expert occupational physicians identify the potential occupational activities and exposures potentially causative, and assign an association strength (imputability) to each one of the exposures. For each patient, are also registered into the database the socio-demographic information as well as clinical results. As a result, the anonymized database contains about 200,000 observations on environmental or work-related diseases [10] and is available on request for the different active network members (physicians and researchers). Table 1 provides the correspondence between variables described in SA in Fig 1 (“Data preparation”) and those from the RNV3P database.

**Table 1:** Correspondence between SA variables and variables in the RNV3P database.

General spectral analysis	Correspondence in RNV3P
Target Variable	Occupational disease
*VE1*, *VE2…*	*NHL (ICD-10 code*: *C82X*, *C83X and C85X)*
Explanatory variables related to the target	Occupational hazards potentially related to the disease
*Modalities A*, *B*, *C*, *D…*	*Name of occupational exposures potentially related to NHL* *(Example*: *“solvents”*, *“pesticides”…)*
Maximum length of nodes	5
Presence of an imputability variable	Yes (0 to 3)

**Fig 2:**
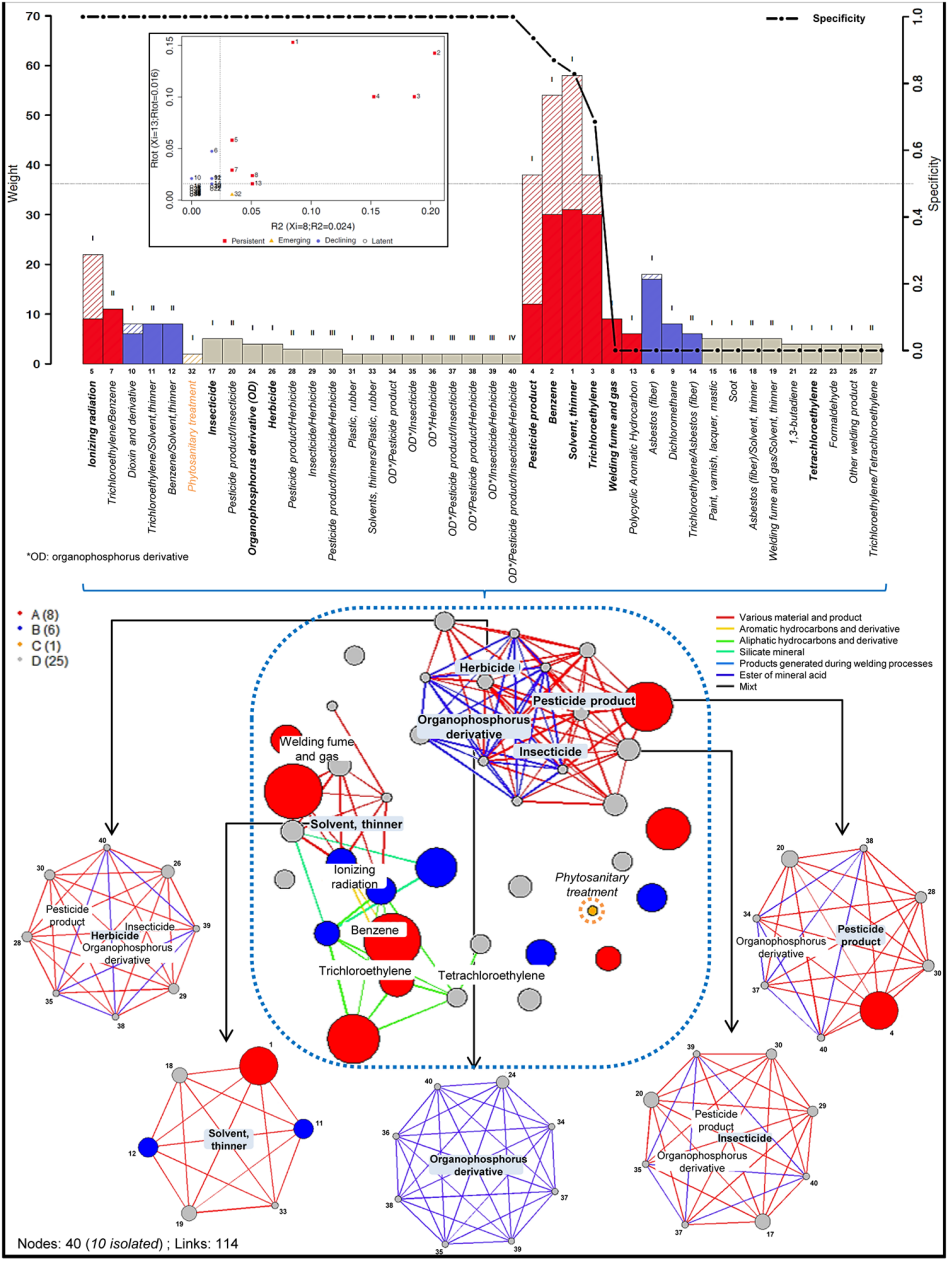
Spectral analysis results for the 40 OEMs related to non-Hodgkin lymphoma from the RNV3P database– 2014. The spectrum for NHL, ranked by specificity, dynamic status, weight and order, is presented at the top of the figure. Each bar corresponds to a significant OEM related to NHL. The size of the bar corresponds to the weight of the OEM, and the color corresponds to the dynamic status. The black curve corresponds to the specificity of each OEM. The insert represents the dynamic status of each OEM according to its reported index values. The spectrosome of NHL, or the relational network of all exposures potentially related to NHL, is presented at the bottom of the figure. The size of the nodes corresponds to the weight of the OEM and the colors correspond to the dynamic status. Motifs that play an important role in the spectrosome structure and have been highlighted on the spectrosome: from left to right: “Herbicide” “Solvent, thinner”, “Organophosphorus derivative”, “Insecticide”, “Pesticide product”.

In 2014, 288 cases of work-related NHL were extracted, including 251 men and 37 women. The first analysis highlighted 178 hypothetical occupational exposures identified by RNV3P physicians as potentially related to NHL, mostly organic chemicals (aliphatic or aromatic hydrocarbons) and industrial substances such as pesticides. Using the SA, the aim was to determine which modalities are more strongly potentially related to NHL, as exposures or combinations of exposures potentially related to NHL, called “occupational exposure motifs” (OEM). Based on the imputability assigned by physicians for each exposure (criterion 1 in Fig 1), 200 of the initial observations have been kept for further analysis. In 2014, after selection and construction from the 178 initial exposures, 40 OEMs have successfully passed the criterion 2 (Fig 1) and were highlighted to constitute the exposure spectrum of work-related NHL (top of the Fig 2 and Table 1).

Each OEM is represented on the spectrum as a bar and ranked by the four hierarchical levels: specificity, dynamic status, weight and order described in the method section. For NHL, among the 40 OEMs identified and listed in Table 2, 21 of them were single exposures (order I) and 19 were combinations of exposures: 13 were combinations of two exposures (order II), 5 were combinations of three exposures (order III), and 1 was a combination of four exposures (order IV). Principal OEMs mostly registered and related to NHL were general substances “solvents, thinners” with a weight of 58 cases, and specific substances such as “benzene” (n = 54) and “trichloroethylene” (n = 38), “pesticide product” (n = 38) and “ionizing radiation” (n = 22).

**Table 2:** List of the 40 OEMs potentially related to NHL from the RNV3P database– 2014.

ID	Order 1	Order 2	Order 3	Nodes	Weight	Status*
**1**	**solvents, thinners**	**-**	**-**	**34**	**58**	**A**
**2**	**benzene**	**-**	**-**	**33**	**54**	**A**
**3**	**trichloroethylene**	**-**	**-**	**30**	**38**	**A**
**4**	**pesticide products**	**-**	**-**	**12**	**38**	**A**
**5**	**ionizing radiation**	**-**	**-**	**10**	**22**	**A**
**6**	**asbestos (fiber)**	**-**	**-**	**18**	**18**	**B**
**7**	**trichloroethylene**	**benzene**	**-**	**10**	**11**	**A**
**8**	**fumes and welding fumes**	**-**	**-**	**9**	**9**	**A**
**9**	**dichloromethane**	**-**	**-**	**8**	**8**	**B**
**10**	**dioxin and derivatives**	**-**	**-**	**7**	**8**	**B**
**11**	**trichloroethylene**	**solvents, thinners**	**-**	**7**	**8**	**B**
**12**	**benzene**	**solvents, thinners**	**-**	**7**	**8**	**B**
**13**	**PAH**	**-**	**-**	**6**	**6**	**A**
**14**	**trichloroethylene**	**asbestos (fiber)**	**-**	**6**	**6**	**B**
15	paint, varnish, lacquer, mastic	-	-	5	5	D
16	soot	-	-	5	5	D
**17**	**insecticides**	**-**	**-**	**4**	**5**	**D**
18	asbestos (fiber)	solvents, thinners	-	5	5	D
19	fumes and welding fumes	solvents, thinners	-	5	5	D
**20**	**pesticide products**	**insecticides**	**-**	**4**	**5**	**D**
**21**	**1,3-butadiene**	**-**	**-**	**4**	**4**	**D**
22	tetrachloroethylene	-	-	4	4	D
23	formaldehyde	-	-	4	4	D
24	organophosphate derivatives	-	-	3	4	D
25	other welding products	-	-	4	4	D
26	herbicides	-	-	3	4	D
27	trichloroethylene	tetrachloroethylene	-	4	4	D
28	pesticide products	herbicides	-	2	3	D
29	insecticides	herbicides	-	2	3	D
30	pesticide products	insecticides	herbicides	2	3	D
31	plastic material, rubber	-	-	1	2	D
**32**	**phytosanitary treatments**	**-**	**-**	**1**	**2**	**C**
33	solvents, thinners	plastic materials, rubber	-	1	2	D
34	organophosphate derivatives	pesticide products	-	1	2	D
35	organophosphate derivatives	insecticides	-	1	2	D
36	organophosphate derivatives	herbicides	-	1	2	D
37	organophosphate derivatives	pesticide products	insecticides	1	2	D
38	organophosphate derivatives	pesticide products	herbicides	1	2	D
39	organophosphate derivatives	insecticides	herbicides	1	2	D
40	organophosphate derivatives	pesticide products	insecticides	1	2	D

* Status: A—Persistent; B—Declining; C—Emergent; D—Latent

As described in the Dynamic status item of the Method section, the SA allowed to model and evaluate the evolution of each OEM related to NHL over time (Fig 2, top insert). Among the 40 initial OEMs, we found 8 persistents (red in Fig 2) that have been strongly recorded by physicians since 2001, 1 emerging OEM that was significantly recruited for two years (orange in Fig 2), 6 declining OEMs with a small recruitment during the last two years (blue in Fig 2) and 25 OEMs to survey in the latency state (gray in Fig 2). However, among the 25 latent OEMs, 3 of them were in an emerging state in 2013 (id: 17, 20 and 21).

According to the specificity indicator, 22 OEMs were characterized as specific and recorded mostly in the same combinations of exposures, but 16 of them were latent OEMs, which were only recruited a few times. However, two of them were persistent OEMs (id: 5 and 7). For example, “ionizing radiation” was recorded 22 times in 10 different combinations of exposures, including one that accounted for 13 recordings (Table 2 and Fig 2). In contrast, 14 of the OEMs were found to have a specificity equal to 0, involving a ubiquitous status for these OEMs. Two of these ubiquitous OEMs were persistent (id: 8 and 13), with a perfect distribution of their frequency among several combinations of exposures: 9 recruitments in 9 distinct combinations of exposures for “fumes/welding fumes” and 6 recruitments in 6 distinct combinations of exposures for “polycyclic aromatic hydrocarbon” (Table 2 and Fig 2).

As explained above, the NHL spectrum allowed highlighting and characterizes each event of interest, and it represents a compendium of the spectrosome that enables the provision of the structure and relationships between the identified OEMs. For the spectrosome of occupational exposures potentially related to NHL (Fig 2, middle), two important types of exposures have been identified: industrial substances (such as solvents, fumes and radiation) and pesticide products (as herbicides, insecticides and organophosphates). These types of exposures were clearly separated on the network and any connections were made between them, leading to the consideration of two very different workplaces and conditions leading to the development of the same pathology. Globally, ten clusters were identified: “pesticide products” (general family), “organophosphorus derivatives”, “insecticides”, “herbicides”, “solvents, thinners”, “benzene”, “trichloroethylene”, “tetrachloroethylene”, “ionizing radiation” and “welding fumes and gas”. Five of these clusters were relatively important in the NHL spectrosome structure due to the important number of links they constituted (Fig 2, bottom). All of these clusters have not been highlighted by an important number of recruitment in Table 2, but from the point of view of structure, they represented a major part of the spectrosome construction and illustrated the multi-exposure potentially related to an occupational disease such as NHL. Among the 40 OEMs, 10 of them were not linked to the others: “ionizing radiation”, “dichloromethane”, “dioxin and derivatives”, “PAH”, “soot”, “1.3-butadiene”, “formaldehyde”, “welding products” and “phytosanitary treatment”. These isolations translate into a non-interaction with another exposure, then a specific condition of exposure on the workplace. The “phytosanitary treatment” OEM was the only one of the occupational exposure OEMs that was found to be emergent in 2014 (orange in Fig 2).

**Fig 3:**
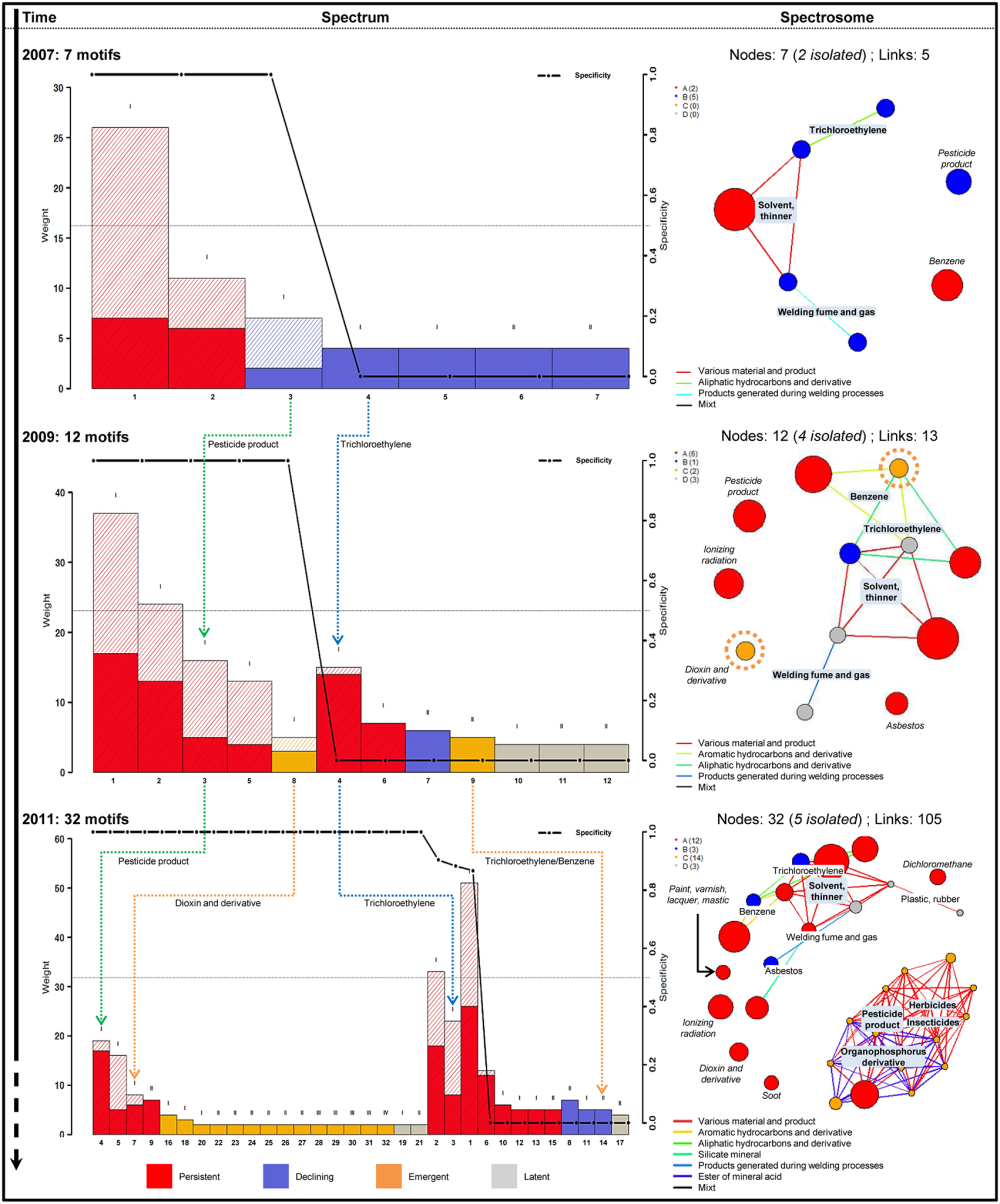
The evolution of spectral analysis results for NHL—2007, 2009 and 2011. Each spectrum and spectrosome of NHL for 2007, 2009 and 2011 are presented from the left to the right, from the top to the bottom. Particular examples have been highlighted to illustrate the evolution: “pesticide products” in green, “trichloroethylene” in blue and the “dioxin and derivatives” and “trichloroethylene/benzene” association in orange.

Fig 2 corresponds to the state of knowledge about NHL in 2014, but knowledge is constantly evolving and surveillance is a continuous process of collecting and analyzing data over time, allowing for evolution in the interpretation. To illustrate the surveillance and the detection of events of interest over time with the SA approach, the spectra and spectrosomes of NHL in 2005, 2007 and 2011 are presented in Fig 3. Each spectrum and spectrosome represents the current state of knowledge for the considered year, and synthesis of the evolution of each OEM is available in Supplemental S1 Table.

In 2007 (Fig 3, top), 76 cases NHL cases were recorded, with a panel of 68 distinct occupational exposures. Only seven occupational exposure OEMs were highlighted, including two of order II: “solvent, thinner/trichloroethylene” and “solvent, thinner/welding fumes and gas”, and two OEMs were isolated, “benzene” and “pesticide products”. The most important OEM in 2007 was “solvent, thinner” with 26 recordings, following by “benzene” (n = 11); these two OEMs were the only ones with a persistent status (red).

In 2009 (Fig 3, middle), 140 NHL cases were extracted for a panel of 102 potential distinct occupational exposures. Twelve OEMs were identified, including 3 of order II, the same OEMs as in 2007 and a new one: “trichloroethylene/benzene”, which was also an emergent OEM (orange). Six OEMs were persistent (red), including “solvent, thinner” and “benzene” which were also persistent in 2005; two were emergent (orange); one was declining (“trichloroethylene/solvents, thinners”, in blue) and 3 were in a latent state (gray). Four OEMs were isolated, “pesticide product”, “ionizing radiation”, “asbestos” and “dioxin and derivatives”, which was also an emergent OEM in 2009 with 5 recordings.

In 2011 (Fig 3, bottom), there were 196 cases of NHL recorded in the RNV3P database and 196 potential occupational exposures. Thirty-two OEMs were highlighted, including 10 OEMs of order II, 4 OEMs of order III and 1 OEM of order IV, and 5 isolated OEMs: “dichloromethane”, “paint, varnish, lacquer, mastic”, “ionizing radiation”, “dioxin and derivatives” and “soot”. The OEM “dioxin and derivatives”, which was emergent and isolated in 2009, was found to be persistent and still isolated in 2011; “ionizing radiation” was also still isolated. In contrast, “trichloroethylene/benzene" was found to be declining. There was a large emergence of a cluster composed of “pesticide products” as a general family and “herbicides”, “insecticides” and “organophosphorus derivatives”, involving an important complexification of data, and the appearance of clusters highlighted in 2014 in Fig 2.

The application of an annual analysis enables the detection of the emergence of events of interest and the monitoring of these events over time. For example, of “dioxin and derivatives” and “trichloroethylene/benzene” were emergent in 2009 and then persistent and declining, respectively, in 2011 (orange arrows). We can also see that due to the specificity indicator, for a persistent OEM, there are several types of recruitment. For example, “pesticide product” (green arrow) and “trichloroethylene” (blue arrow) were different in terms of recruitment with an important specificity for the “pesticide product” OEM and a high diversity for the “trichloroethylene” OEM.

## Discussion

The aim of this study was to develop an approach enabling an optimal exploitation of databases, analyzing, characterizing, describing, and, therefore, improving our understanding of occupational environment-health relationships. We have developed a spectral analysis approach to characterize the dynamic natures of occupational health problems (OHPs) and their associations. The main objective of the SA approach is to enable an optimal use of large-scale observational databases, taking into account time and multiplicity of causes leading to the appearance of an event of interest. To demonstrate the spectral analysis procedure, we used the non-Hodgkin lymphoma (NHL) sample from the RNV3P database to study the dynamics of pathology-occupational exposure associations as “OEMs”, analyze the structural changes in these associations, and highlight the appearance or disappearance of OEMs.

For NHL, 40 OEMs were highlighted in 2014, and 18 of them were active (persistent, emergent or in decline) (Table 1 and Fig 2). An important multi-exposure related to NHL was shown with a total of 19 complex OEMs (47.5% of OEMs). The most important OEMs identified were solvents and thinners (including benzene and trichloroethylene), asbestos and pesticide products (including herbicides and insecticides at a lower level). Some exposures were also highlighted as “isolated”, referring to specific workplaces where there is no interaction between exposures or where risk factors are correctly identified. The majority of OEMs highlighted by spectral analysis are consistent with findings in the literature. For example, Rieutort et al. ranked occupational exposures mentioned in the literature from 1990 to 2013, and there are 14 perfect matches with the SA results on the 91 occupational exposures mentioned in the article and the 49 OEMs highlighted in the SA [17]. The main difference is that the SA takes multi-exposure into account (and some OEMs are a combination of exposures) and the literature does not. For example, the combination of welding fumes and solvents was highlighted in the SA approach but not mentioned as a combination in the ranking of occupational exposures. Several exposures from the literature were not considered as OEMs, but this does not mean that these exposures do not exist in the RNV3P database, but rather that they did not pass the criterion of significance.

Furthermore, it is also possible to use the other descriptive variables in the input file to better describe the relationship between the target and explanatory variables. For example, in the RNV3P database, there is information about the occupational activities of each patient. Thus, it could be interesting to cross OEM information with occupational activities to define the riskiest activities. For example, the occupational activities with the most recorded OEMs related to NHL were “farmers and commercial agriculture workers” in the “crop and animal production, hunting and related services” sector; and “specialist in physical, mathematical and engineering sciences” in the “chemical industry” sector, with 18 and 19 OEMs, respectively, mostly recorded in 2010 and 2011.

The analysis of observational databases as presented addresses another challenge and has the potential to improve knowledge about occupation–health relationships. Unfortunately, statistical tools are limited in their application to those types of databases in which the denominator and the reference population are not defined. Therefore, classical incidence and prevalence calculations are not suitable. Moreover, the multifactorial aspect is hardly taken into account. Therefore, the generation of a dynamic spectrum from observational data using the SA approach could be a potential solution. Indeed, Fig 3 highlights the usefulness of the update over time, showing the evolution of structure and knowledge about work-related NHL cases. In 2007, only 7 OEMs were highlighted, whereas 40 OEMs were highlighted in 2014, with 5 and 114 links, respectively, on the spectrosomes (Figs 2 and 3). Based on this observation, it could be interesting to use the spectral analysis approach as a new surveillance methodology applied to observational databases. Such an approach could be called “observational spectral analysis” (OSA). Spectral analysis enables the detection of the emergence of events of interest and the monitoring of these events over time, as shown in the results, due to the generation of a dynamic spectrum from observational data.

The OSA approach outlined above is designed for analyzing observational databases by providing a different reading of the information already therein. In this respect, a limitation of the method lies in the sensitivity of the final OSA outcomes to the quality of input data. This could happen because, for instance, the associations between exposures and diagnosed disease are selected and recorded into the database by expert physicians and those associations are subjected to vary depending on expert knowledge and patients during the occupational interview. A way of circumventing this in OSA is to deal with larger number of cases and rerun OSA with varying parameters in a sort of sensitivity analysis.

The approach outlined above is general and goes beyond conventional methods. It could be interesting to apply this approach to other observational databases for surveillance, using 3 mandatory variables as analysis criteria: target, explanatory variable(s) and time variables. The OSA could be used on observational databases for the surveillance of OHPs, the detection of emerging OHPs and the prevention of threats to workers’ health.

## Supporting information

S1 Fig Another simple illustration of motif construction from a set of three nodes. This additional illustration shows the construction of each combination, with 3 nodes containing 5, 3 and 2 modalities, respectively, with weights equal to 2, 4 and 8, respectively. From each node, combinations were successively generated (order I, order II, …). Finally, each distinct combination was identified and attributed a final weight, corresponding to the sum of the weight of each node from which they were generated.
(PDF)

S2 Fig Illustration of the node repartition for a specificity equal to 1 or 0, corresponding to *H*_*m*,*min*_ and *H*_*m*,*max*_ respectively. (PDF)

S1 Table Synthesis of the evolution of each OEM associated with NHL in 2007, 2009, 2011 and 2014. (PDF)
