# Supplementary figures and images for: The spectrosome of occupational health problems
Source: PLoS One. 2018 Jan 5;13(1):e0190196. doi: 10.1371/journal.pone.0190196 (PMC5755768; doi:10.1371/journal.pone.0190196)

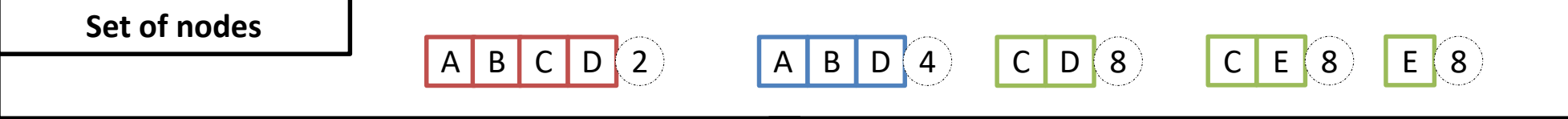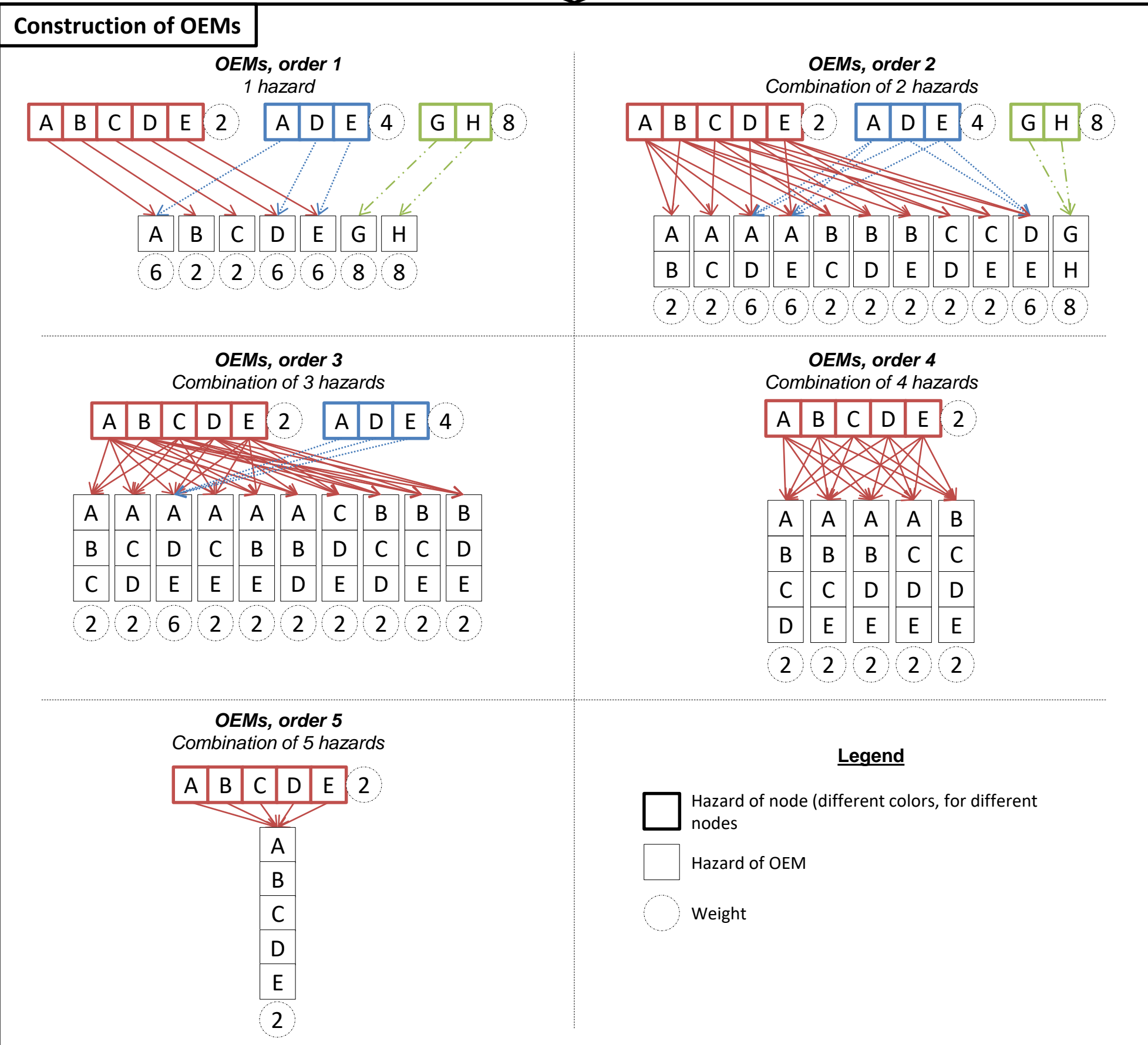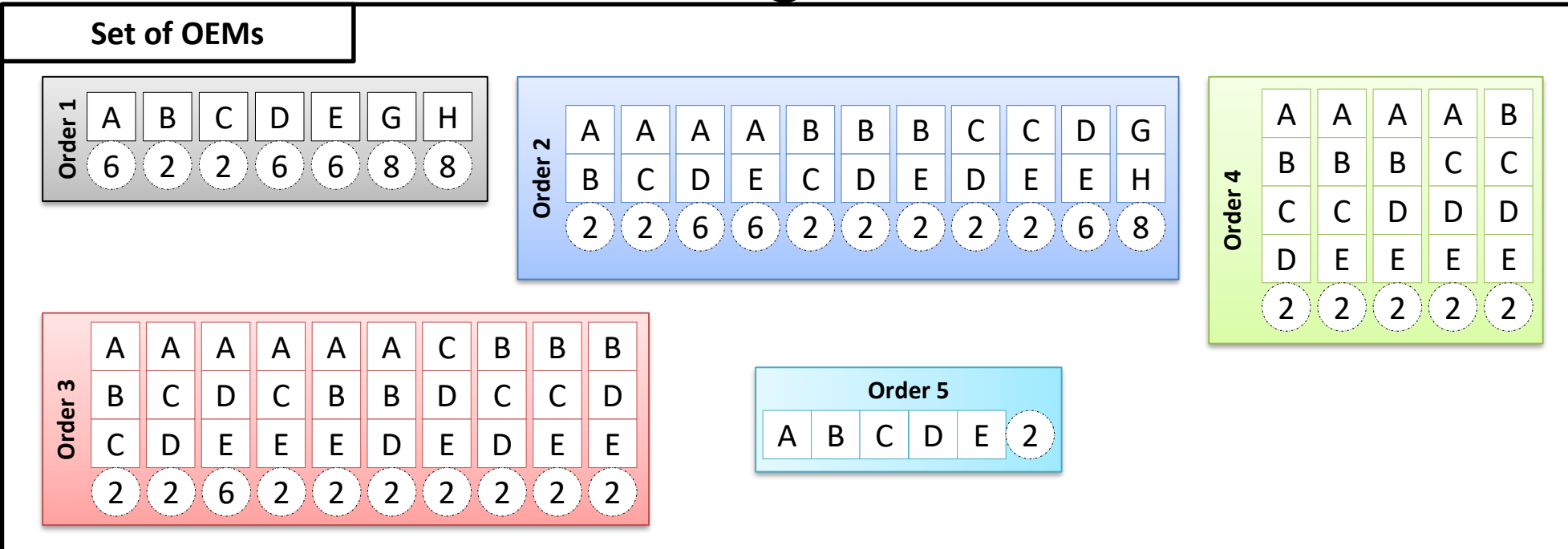

Supplement: S1 Fig — This additional illustration shows the construction of each combination, with 3 nodes containing 5, 3 and 2 modalities, respectively, with weights equal to 2, 4 and 8, respectively. From each node, combinations were successively generated (order I, order II, …). Finally, each distinct combination was identified and attributed a final weight, corresponding to the sum of the weight of each node from which they were generated. (PDF) [file pone.0190196.s001.pdf]

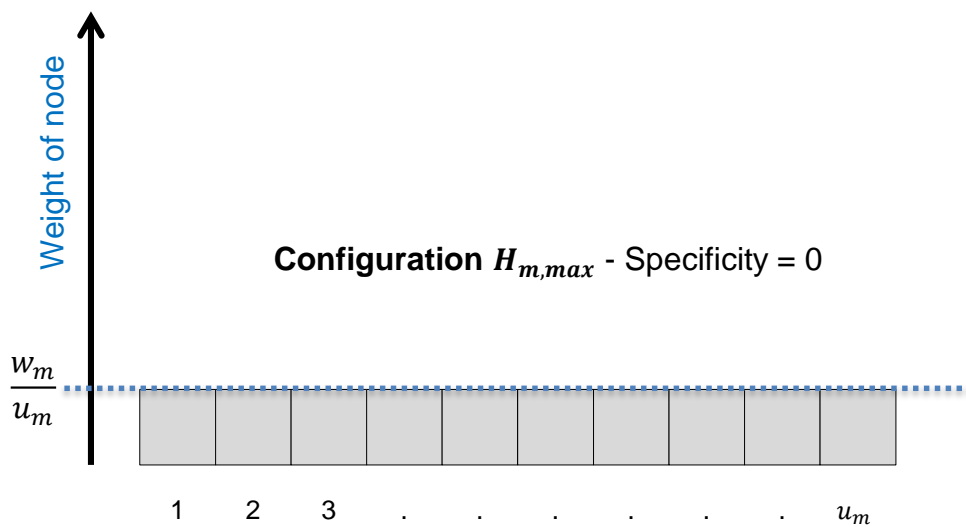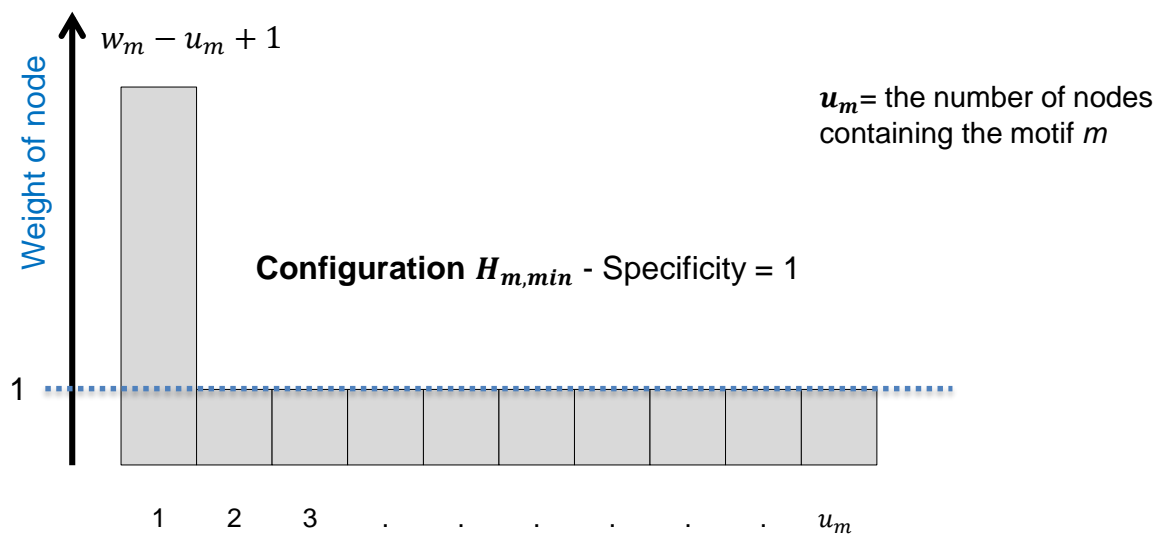

Supplement: S2 Fig — (PDF) [file pone.0190196.s002.pdf]
