# Supplementary material for: The spectrosome of occupational health problems
Source: PLoS One. 2018 Jan 5;13(1):e0190196. doi: 10.1371/journal.pone.0190196 (PMC5755768; doi:10.1371/journal.pone.0190196)
Supplement: S1 Table — (PDF) [file pone.0190196.s003.pdf]

S1 Table: Synthesis of the evolution of each OEM associated with NHL in 2007, 2009, 2011 and 2014.

				2007		2009		2011		2014	
**Order I**	**Order II**	**Order III**	**Order IV**	**ID**	**Status**	**ID**	**Status**	**ID**	**Status**	**ID**	**Status**
**Solvent, thinner**	**-**	**-**	**-**	1	A	1	A	1	A	1	A
**Benzene**	**-**	**-**	**-**	2	A	2	A	2	A	2	A
**Trichloroethylene**	**-**	**-**	**-**	4	B	4	A	4	A	3	A
**Pesticide product**	**-**	**-**	**-**	3	B	3	A	3	A	4	A
Ionizing radiation	-	-	-	-	-	5	A	5	A	5	A
Asbestos (fiber)	-	-	-	-	-	6	A	6	A	6	B
Trichloroethylene	Benzene	-	-	-	-	9	C	14	B	7	A
Welding fume and gas	-	-	-	5	B	10	D	11	B	8	A
Dichloromethane	-	-	-	-	-	-	-	10	A	9	B
Dioxin and derivative	-	-	-	-	-	8	C	7	A	10	B
Trichloroethylene	Solvent, thinner	-	-	6	B	7	B	8	B	11	B
Benzene	Solvent, thinner	-	-	-	-	11	D	9	A	12	B
PAHs	-	-	-	-	-	-	-	-	-	13	A
Trichloroethylene	Asbestos (fiber)	-	-	-	-	-	-	-	-	14	B
Paint, varnish, lacquer, mastic	-	-	-	-	-	-	-	12	A	15	D
Soot	-	-	-	-	-	-	-	13	A	16	D
Insecticides	-	-	-	-	-	-	-	20	C	17	D
Asbestos (fiber)	Solvent, thinner	-	-	-	-	-	-	15	A	18	D
Welding fume and gas	Solvent, thinner	-	-	7	B	12	D	17	D	19	D
Pesticide product	Insecticides	-	-	-	-	-	-	24	C	20	D
1,3-butadiene	-	-	-	-	-	-	-	-	-	21	D
Tetrachloroethylene	-	-	-	-	-	-	-	-	-	22	D
Formaldehyde	-	-	-	-	-	-	-	-	-	23	D
Organophosphorus derivative (OD)	-	-	-	-	-	-	-	16	C	24	D
Other welding product	-	-	-	-	-	-	-	-	-	25	D
Herbicides	-	-	-	-	-	-	-	18	C	26	D
Trichloroethylene	Tetrachloroethylene	-	-	-	-	-	-	-	-	27	D
Pesticide product	Herbicides	-	-	-	-	-	-	26	C	28	D
Insecticides	Herbicides	-	-	-	-	-	-	27	C	29	D
Pesticide product	Insecticides	Herbicides	-	-	-	-	-	31	C	30	D
Plastic, rubber	-	-	-	-	-	-	-	19	D	31	D
Phytosanitary treatment	-	-	-	-	-	-	-	-	-	32	C
Solvent, thinner	Plastic, rubber	-	-	-	-	-	-	21	D	33	D
OD*	Pesticide product	-	-	-	-	-	-	22	C	34	D
OD*	Insecticides	-	-	-	-	-	-	23	C	35	D
OD*	Herbicides	-	-	-	-	-	-	25	C	36	D
OD*	Pesticide product	Insecticides	-	-	-	-	-	28	C	37	D
OD*	Pesticide product	Herbicides	-	-	-	-	-	29	C	38	D
OD*	Insecticides	Herbicides	-	-	-	-	-	30	C	39	D
OD*	Pesticide product	Insecticides	Herbicides	-	-	-	-	32	C	40	D

** Organophosphate derivatives*
